# The Activation and Selectivity of the *Legionella* RavD Deubiquitinase

**DOI:** 10.3389/fmolb.2021.770320

**Published:** 2021-11-16

**Authors:** Eric Schulze-Niemand, Michael Naumann, Matthias Stein

**Affiliations:** ^1^ Molecular Simulations and Design Group, Max Planck Institute for Dynamics of Complex Technical Systems, Magdeburg, Germany; ^2^ Medical Faculty, Institute for Experimental Internal Medicine, Otto-von-Guericke University, Magdeburg, Germany

**Keywords:** substrate-assisted activation, catalytic mechanism, deubiquitinating enzymes, X-ray structures, protein-protein complex, structural biology

## Introduction

Deubiquitinating enzymes (DUBs) have critical regulatory roles in the ubiquitin system through their ability to specifically deconjugate ubiquitin protein molecules from targeted proteins. Their linkage selectivity is only marginally rationalized and an understanding of these cellular processes is important since DUBs have recently emerged as therapeutic targets against cancer (Shi and Grossman, 2010).

The “substrate-assisted catalysis” (SAC) is a relatively novel concept for the activation of an enzyme (Dall’Acqua and Carter, 2000). Here, a functional group or a moiety of the substrate molecule itself interacts with the enzyme’s active site and leads to an activation procedure, e.g. a conformational re-arrangement or a deprotonation event.

OTULIN is the only human DUB with an exclusive linkage selectivity towards methionine-1 (M1) linked di-ubiquitins. Two distinct ubiquitin recognition sites S1 (proximal) and S1’ (distal) form contacts with the substrate. OTULIN binding to di-ubiquitin is characterized by a substrate-assisted catalysis step, in which the catalytic triad is only activated upon tight substrate binding (Keusekotten et al., 2013). The proximal ubiquitin residue Glu16 inserts into the catalytic center and stabilizes a conformational state in which the mobility is restricted so that the catalytic residues are properly aligned, and the enzyme becomes activated.

Various pathogens from bacteria and viruses have independently evolved proteins that mimic human ovarian tumor domain (OTU) deubiquitinases in order to circumvent an innate immune response (Schlüter et al., 2021). For example, recently a deubiquitinase from *Legionella pneumophila*, RavD, was characterized (Wan et al., 2019). Analysis of structural data and enzyme assays clearly resolved its DUB activity. In particular, RavD shares the exclusive M1-linkage specificity with the human ovarian tumor domain protease OTULIN. RavD is a papain-like deubiquitinase with a characteristic Cys–His–Ser catalytic triad and exhibits an overall structure that is dissimilar to OTULIN and may thus be considered the founding member of a novel class of DUBs (Hermanns and Hofmann, 2019). Unlike OTULIN, there were no large conformational changes reported, when comparing the X-ray structures of only the RavD and when in complex with the di-ubiquitin substrate.

The authors reach the conclusion that M1-linkage selectivity of RavD results only from the two distinct ubiquitin recognition sites S1 (distal) and S1’ (proximal). Due to the absence of a large conformational re-arrangement of the active site residues upon ubiquitin-binding, they explicitly rule out that the catalytic mechanism of RavD is assisted by ubiquitin. The authors used a non-hydrolysable substrate-analogue in the RavD di-ubiquitin co-crystal structure which retains a large distance between the scissile bond and the nucleophile and the catalytic triad remains in an inactive state.

## Results

### Substrate-Assisted Activation of Human OTULIN

The prime criterion for a clear identification of a substrate-assisted enzymatic reaction mechanism on OTUs and DUBs is the analysis of the structural arrangement within the catalytic center between the unbound enzyme and the enzyme-substrate complex (Mevissen and Komander, 2017).

For human OTULIN, a detailed comparison of the protein structures of the pure enzyme (PDB entry 3ZNV) and when in complex with the di-ubiquitin substrate (PDB entry 3ZNZ) allows the investigation of structural changes upon substrate binding (Keusekotten et al., 2013). Whereas no large global protein conformational changes can be detected, inter-residue distances of the active site undergo remarkable reductions. In the inactive (apo) state, residue distances of 6.5 and 8.3 Å are indicative of an “inactive” conformation.

**FIGURE 1:**
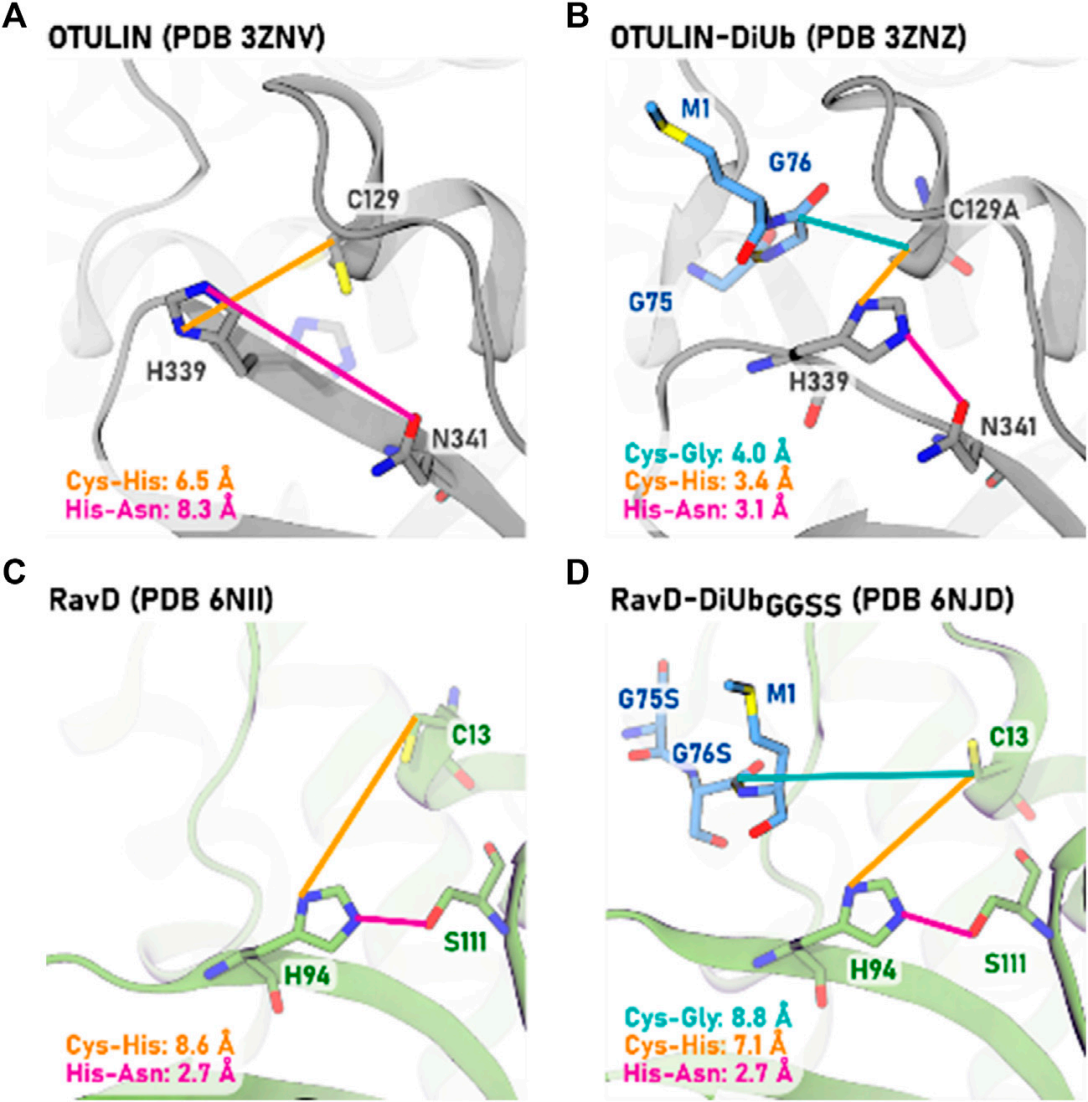
Comparison of structural parameters of catalytic triad residues in methionine 1-specific DUBs OTULIN [**(A,B)**, gray] and RavD [**(C,D)**, green]. The apo-protein structures **(A,C)** are compared to the protein-protein structures when in complex with their di-ubiquitin substrate **(B,D)**. The PDB entries are given in brackets. The distances to cysteine are measured from the residue’s Cβ atom for reasons of comparability with the OTULIN-diubiquitin crystal structure in which cysteine was mutated to alanine (C129A). For apo-OTULIN, an alternative conformation of His339 with lower occupancy is also present (shown in transparent), which is identical to the conformation of His339 in OTULIN-DiUb complex.

Upon substrate binding, the active site reaches a reactive conformation: the scissile bond comes as close as 4.0 Å to the cysteine, and the inter-residue distances for the catalytic triad residues decrease from 6.5 to 3.4 Å (Cys-His) and from 8.3 to 3.1 Å (His-Asn), Figure 1. Only this tight complex allows the deprotonation of cysteine by histidine and thereupon the activation of the catalytic triad the formation the zwitterionic state (Cys^−^/His^+^).

### The Structural Biology of RavD Pathogen Activation

RavD shares the exclusive di-ubiquitin M1-linkage selectivity with human OTULIN, but different steps of activation were reported (Wan et al., 2019). Protein structures of the apo (PDB entry 6NII) and the substrate-bound enzyme (PDB entry 6NJD) revealed the binding mode of linear methionine-1 linked di-ubiquitin to RavD to be almost identical to the one in OTULIN.

For RavD in the absence of substrate, also those large active site inter-residue distances can be seen (Figure 1). Compared to OTULIN, however, also in the RavD-substrate crystal structure catalytic inter-residue distances remain as large as 8.8 Å for the scissile bond to cysteine, and 7.1 Å (for Cys-His) and 2.7 Å (His-Ser). This is beyond a reactive distance for a cysteine protease (Buller and Townsend, 2013). This absence of structural re-arrangements led the authors to rule out a substrate-assisted activation step and they assigned the origin of the M1-selectivity to only the ubiquitin binding sites S1 and S1’.

It has to be noted that the co-crystal structure of di-ubiquitin RavD was obtained when complexed with a non-hydrolysable di-ubiquitin substrate analogue in which the two terminal glycine residues of the distal ubiquitin are mutated to serine residues (diUb_GGSS_). We argue that the introduction of those larger and polar serine residues obstructs full substrate access to the active site. They form strong interactions with RavD surface residues Gln42 and Ala92, which hinders full substrate insertion into the catalytic groove, and hence the essential conformational change of the catalytic triad.

## Discussion

The state of activation of the catalytic site is critical for its role in cell signaling and the discovery of selective inhibitors. As an example, we refer to the human OTUs OTUB1 and OTUB2. They both display the characteristic Cys-His-Asp/Asn catalytic triad, but differ in their active site residues’ protonation states and conformations (Sivakumar et al., 2020). In the absence of ubiquitin, OTUB1 is in an inactive conformation but substrate binding induces a conformational change and movement of His265 towards Cys91. The active site in OTUB2, however, is already in a pre-arranged, catalytically competent conformation prior to ubiquitin-binding. This mechanistic difference explains the high selectivity of OTUB1 towards K48-linked ubiquitin and the broad substrate promiscuity of OTUB2. The catalytically competent active site, even in absence of substrate, may be one of the reasons for the successful design of selective covalent small-molecule inhibitors for OTUB2 from electrophile fragment screening (Resnick et al., 2019).

The molecular basis for DUB specificity is only emerging recently and requires careful comparison of structural details. Mono-ubiquitin protein co-crystals depict the product rather than a substrate-bound form and cannot explain DUB selectivity. Only when crystallized with its substrate Lys11-di-ubiquitin, the structural basis for the DUB Cezanne linkage selectivity in terms of the S1’ site and conformational changes around the catalytic center upon substrate binding could be explained (Mevissen et al., 2016).

For the generation of co-crystals of RavD with diubiquitin (diUb) substrate, the authors used the above described non-hydrolysable substrate analogue. Based on their crystal structures, the authors reached the conclusion that M1-linkage selectivity of RavD results only from two distinct ubiquitin recognition sites S1 (distal) and S1’ (proximal). The absence of structural re-arrangements of the catalytic triad upon substrate binding has led to the disregard of the concept of substrate-assisted catalysis in RavD. This, however, cannot rationalize the common substrate selectivity. It is suggested that there is no full substrate access in RavD to the active site due to the use of a two-fold modified substrate analogue.

An unambiguous assignment as to the origin of M1-linkage selectivity of RavD can only be obtained when the substrate co-crystallizes in a catalytically competent conformation. We suggest that, instead of mutating residues of the methionine 1-linked di-ubiquitin substrate, a modification of the proteolytic cysteine residue into a non-reactive alanine could give a more conclusive structure close to the physiological state.

The assignment and identification of an enzyme activation mechanism is critical when aiming at a selective targeting of a pathogen, here from *Legionella*, but not its human equivalent.
